# Personalised Medicine and the Economy of Biotechnological Promise

**DOI:** 10.1080/20502877.2017.1314892

**Published:** 2017-05-18

**Authors:** Steve Sturdy

**Affiliations:** ^a^ Science, Technology and Innovation Studies, University of Edinburgh, Edinburgh, UK

**Keywords:** personalised medicine, promissory economy, biotechnology, medical innovation, drug discovery, genomics

## Abstract

Rather than seek to distinguish hype from legitimate promise, it may be more helpful to think about personalised medicine as embodying a promissory economy which serves both to mobilize resources for research and — partly at least — to determine the ends to which that research is directed. Personalised medicine is a development of the larger promissory economy of medical biotechnology. As such, it systematically conflates public benefit with the pursuit of commercial and especially pharmaceutical interests. Consequently, research and development in personalised medicine tends to favour the production of expensive new treatments over unprofitable forms of prevention or more effective use of older therapies. A rebalancing of research priorities is needed to favour the pursuit of public benefit, even when it does not deliver private profits. This will in turn require sustained reflection, self-criticism and often self-denial on the part of public research funders and the scientists they support.

Science is driven by promises. Without the promise of edifying knowledge, useful new technologies, and other social goods, scientists would not devote their careers to research, nor society provide the very substantial resources that modern science consumes. Yet the outcomes of scientific research are by their nature uncertain and unpredictable. Consequently, much of what science promises inevitably goes unfulfilled, while what scientists actually deliver is frequently different from what was promised.

This is not necessarily a problem. Society is often willing to overlook scientists’ failure to deliver on particular promises, so long as science is seen as yielding social benefit overall. At times, however, the mismatch between promise and delivery comes to be seen as problematic, be it by scientists or their funders or the wider public. At such times, it is tempting to suppose that the problem lies in the dissemination of unrealistic or irresponsible promises — of ‘hype’, in other words — and that the best response is to try and restrict the circulation of scientific promises to include only those with a realistic hope of fulfilment. Such an approach is likely to be self-defeating, however. Any attempt to distinguish those promises which are likely to be realised from those which are likely to fail will founder on the uncertainties inherent in scientific research and development, and runs the risk of foreclosing on unforeseen advances. Ultimately, hype can only be definitively distinguished from promise with the privilege of hindsight — by which time it will be too late to be useful.

That does not mean that scientific promise is beyond meaningful scrutiny. Rather, it means that we need to adopt a different way of thinking about it. Instead of trying to predict the future, it may be more helpful to focus on the present. And instead of asking whether promises are likely to come true, it may be more helpful to ask what function they serve. Promises are part and parcel of the social relations of science. Not least, they are a vital element in the economy of science. As such, they need to be seen in relation to the flow of resources and goods that sustains and to an extent determines the nature of scientific work. What kind of promises circulate at any given time, what degree of credibility they enjoy, what resources they help to mobilize, and what consequences follow from their fulfilment or non-fulfilment, all depend upon the structure and dynamics of the particular scientific field in which they arise. Promises are shaped by the aims and interests of the various individuals, groups and institutions who make up that field, including the scientific knowledge and other goods that they seek to deliver, as well as the resources and rewards they hope to garner. In short, scientific promises are best understood in the context of particular promissory economies (e.g. Brown *et al*. 2000; Martin 2015). Once we adopt this point of view, we can begin to ask other questions about scientific promises besides whether they are likely to come true or not. We can ask why particular kinds of scientific promises are being articulated and circulated, why resources are being devoted to fulfilling particular problems and not others, and what this means for the direction in which science is developing.

This approach can help to throw light on current anxieties that the promise of personalised medicine has become tainted by hype. Precisely what counts as personalised medicine is a matter of debate. But most current definitions focus on the development and implementation of tests for biomarkers which either indicate elevated risk of developing certain conditions or which identify individuals most likely to benefit from particular therapeutic and especially pharmaceutical interventions. Research in personalised medicine thus tends to focus on identifying biomarkers, including but by no means confined to genomic markers; elucidating how those biomarkers relate to health status, drug response etc.; and developing interventions aimed at preventing or treating the illnesses with which those markers are associated (Tutton 2014). This definition of personalised medicine serves to locate it in a quite specific social and historical context. Personalised medicine can be seen as an expression of the medical biotechnology sector as it developed from the 1970s onwards. Consequently, if we want to understand current concerns about the promise and hype of personalised medicine, we might usefully examine the evolution of the promissory economy of medical biotechnology.

The emergence of the biotechnology sector from the mid-1970s coincided with and helped to catalyse a major reconfiguration of the economy of scientific promise. Before the 1960s, public support for science was rarely accompanied by demands for evidence of specific social benefits. On the contrary, it was widely accepted that leaving ‘basic’ science to proceed unhindered by the need to engage directly with societal needs was the best way to ensure that it would ultimately lead to useful if often unforeseen applications. By the 1970s, however, a combination of factors, from left-wing critiques of the way that science was linked to military purposes, to right-wing concern at burgeoning government science budgets, led to new demands for more tangible evidence that science did indeed deliver public benefit. For many, particularly in the USA, the delivery of public goods was increasingly understood in terms of the commercial development of new commodities and services, including new medicines and diagnostic services; while national prosperity became identified with the pursuit of innovation-based industrial competitiveness (Gibbons *et al*. 1994; Jasanoff 2005, pp. 225–246; Berman 2015). In effect, the value of science as a public good came at this time to be regarded in large part in terms of its ability to drive commercial activity.

In this context, the demonstration in 1974 that living cells could be genetically engineered to produce proteins encoded by DNA from other species was widely hailed, not just as a major achievement in basic science, but as the beginnings of a revolutionary new production technology with the potential to transform pharmaceutical production, in particular. Private investors began to fund new biotechnology ventures, including both new start-up companies and commercially oriented academic research programmes, particularly in the USA, where measures to encourage venture capital and other forms of speculative investment were being enacted around the same time. By the early 1980s the biotechnology sector was gaining ground as a distinctively novel form of scientific life, characterized by an unprecedented degree of inter-penetration of academic and commercial institutional forms, and by a profound blurring of older distinctions between basic science and market-oriented research and development (de Chadarevian 2011; Rasmussen 2014; Yi 2015; Owen and Hopkins 2016).

Commercial promise was vital to the growth of biotechnology. Given the delays and uncertainties between early-stage scientific research and the eventual delivery of new products to market, the ability to secure private investment depended heavily on generating persuasive indicators of a future return on that investment. Patents had particular promissory value in this regard (Doganova and Muniesa 2015), and steps were taken in a number of countries to make it easier for researchers and universities as well as companies to obtain patents on their work — the USA again leading the way with the passage of the 1980 Bayh–Dole Act among other measures (Berman 2008). Other markers of commercial promise also came to prominence around this time, including ‘cloning by press conference’ — the announcement of scientific results to the press before they appeared in peer-reviewed journals, as much in order to impress investors as to claim scientific priority (Rasmussen 2014, pp. 89–91) — as well as public disclosure of research plans and details of patent applications (Fortun 2008). The biotechnology sector thus enacted a promissory economy in which the funding of research was to a significant extent predicated on the promise, not just of useful new products and services, but of future profits.

The 1980s and 1990s saw rapid expansion not just in the technical capabilities of molecular biology, but in the nature and scope of the biotechnological promises if offered. The first biotechnology companies focused primarily on developing recombinant DNA techniques to manufacture therapeutic molecules such as insulin and interferon. By the mid-1980s, however, researchers had also begun adapting molecular biological methods to the work of mapping and ultimately cloning genes associated with a range of human diseases. Such work initially concentrated on rare single-gene disorders such as Huntington's disease, Duchenne muscular dystrophy and cystic fibrosis. But researchers were optimistic that they would soon be able to identify and clone genes associated with much commoner conditions including cancer and heart disease. This fuelled a further wave of expansion in the promissory economy of biotechnology. As early as the mid-1980s, market analysts were projecting a huge growth in the market for commercial genetic diagnostics, particularly for common disorders (Nelkin and Tancredi 1989, pp. 33–35), and by the early 1990s, hopes were rising for the inception of radically new forms of gene therapy (Martin 1999; Stockdale 1999; Lindee and Mueller 2011).

What most attracted investors, however, was the expectation that research into the molecular genetics of common disorders offered a means of identifying novel molecular targets for new classes of drugs. By the mid-1990s, a second wave of biotechnology startups was emerging which sought to commercialize the means of genomic discovery itself. Companies such as Incyte Pharmaceuticals, Human Genome Sciences, Millennium Pharmaceuticals and later Celera Genomics succeeded in securing not just venture capital, but also lucrative deals with big pharmaceutical companies (Hopkins *et al*. 2007; Gottinger and Umali 2008). Closely linked in many cases to key centres of academic genome research, these initiatives also benefited from the increasing amounts of public and charitable funding devoted to genomic research, from the Human Genome Project to the International Haplotype Mapping Project (International HapMap Consortium 2003) as well as a proliferation of national biobanks. The field of genomics was thus characterized by a remarkable convergence of commercially and publicly funded research.

That funding was in turn underwritten by a promissory rhetoric that further blurred the distinction between public and commercial benefits. Mirroring the rhetoric of first-wave biotechnology, this included talk of the private profits to be made from new diagnostic and therapeutic technologies, as well as the national economic benefits that would come from building thriving biotechnology, pharmaceutical and diagnostics industries. But genomic researchers and their funders also promised other benefits. Appreciation of individual genomic differences would make it possible to assess personal health risks and devise personalised preventive interventions, they argued. It would also ensure that therapeutic measures targeted not just the patient's illness, but also the way they responded to different drugs. By ensuring that patients were prescribed the most suitable drugs for their individual constitution, the new science of pharmacogenetics would also minimize wasteful prescribing of ineffective medicines and reduce the costs of adverse drug reactions (Hedgecoe and Martin 2003; Smart and Martin 2006). It would thus deliver economic savings as well as public health benefits. It was this peculiar combination of preventive and therapeutic promises, with its distinctive alignment of personal health benefits, gains in the economic efficiency of healthcare, and the delivery of profitable new pharmaceutical products, that increasingly came to be publicized under the banner of ‘personalized medicine’ (Tutton 2014).

As of early 2017, fulfilment of these ambitious promises remains patchy at best, as other contributors to this special issue have noted. This is partly because the role of genes in disease aetiology has turned out to be much more complicated than originally envisaged. In only a few cases has it been possible to identify genetic variants that indicate actionable levels of personal risk of common diseases, while even single-gene disorders often involve large numbers of different mutations with widely-varying effects. Consequently, as Kezia Gaitskell observes above, genetic screening has generally proved less effective as a preventive measure than originally envisaged (Gaitskell, this volume).

Rather more success has been achieved in using genomics to illuminate disease pathways, identify druggable targets, and develop new drugs, particularly in cancer and in various auto-immune and inflammatory conditions. Consequently, many of the drugs that have reached the market over the past twenty years owe their discovery in whole or in part to genomic research — though even here, progress has been sporadic (Maughan, this volume). Genomic technologies, including pharmacogenetic stratification of patients, have also been incorporated into the clinical stages of new drug development, with the aim of improving response rates and excluding non-responders from clinical trials. Since many of these new drugs target particular genetic sub-groups of patients, developments in this area might reasonably be regarded as having delivered a degree of ‘personalization’ of medicine. Ironically, however, delivery of such drugs has done little to fulfil the promise that personalised medicine would lead to cost savings. On the contrary, since the patient populations for such drugs are often relatively small, drug companies have generally marketed them at correspondingly high prices, in order to generate the levels of financial return that they expect from new medicines.

Meanwhile, the promise that pharmacogenetic targeting of treatments would deliver greater safety and efficiency in the use of older medications such as warfarin has also gone largely unfulfilled. The reasons for this are complex, including lack of compelling evidence that such targeting is actually beneficial in practice, and reluctance on the part of doctors and healthcare funders to implement new practices in the absence of such evidence (Hedgecoe 2004). However, the unwillingness of pharmaceutical manufacturers to do anything to reduce the size of the patient populations to whom their products are prescribed has also been a significant limiting factor (Danzon and Towse 2002; Hogarth *et al*. 2006).

Seen in economic perspective, personalised medicine has thus been much better at fulfilling its promise to reward private investment, whether in the pharmaceutical or biotechnology sector, than at delivering savings to health services and their funders. This is disappointing. However, once we see the promise of personalised medicine, not just as a prediction of future possibilities, but as part of the promissory economy of biotechnology, it is unsurprising. As we have seen, that economy has grown up around an increasingly close alignment of research, including publicly funded research, with the pursuit of commercial interests; while the fulfilment of biotechnological promise, including the promise of public benefit, is widely assumed to lie in the successful commercialization of that research. Given this privileging of private interests as the means to secure public goods, it is unsurprising that technologies such as pharmacogenetics have chiefly been adopted in ways that are consistent with the pursuit of drug company profits, while applications that might secure economies in healthcare but undermine profitability have largely been ignored.

Meanwhile, substantial public as well as private funds continue to be devoted to supporting research into the genomics of human diseases, and to promoting the commercialization of that research. In the UK, for instance, Genomics England's 100,000 Genome Project combines public and private funding in a bid to elucidate the genetics of cancer and rare diseases, while a similar function is served in the USA by the Cancer Moonshot programme. Such programmes continue to be accompanied by the language of personalization. They are also distinguished by engagement with clinical healthcare, both as a source of patient information and for the opportunities it offers for clinical research. As such, these programmes reflect the desire of funders and researchers to expedite the translation of genomic research findings into medical services and products. The same intentions inform the growth of public and private funding for so-called ‘translational medicine’ — a rather heterogeneous field of research and development activities in which personalised medicine occupies a prominent position; across such initiatives, the idea of translation is commonly conflated with successful commercialization of research (Mittra and Milne 2013). Finally, orphan drug legislation has done much to boost the profitability of biotechnology-based pharmaceuticals aimed at small patient populations (Mikami in preparation). Assisted by such measures, the dominant direction of personalised medicine continues to be towards the production of expensive new treatments for ever-smaller populations of patients.

It is unclear how much longer that trajectory can be sustained. New treatments are constantly straining the limits of what health technology assessment agencies consider cost-effective, while the cumulative cost of such medicines poses a major challenge to even the best-resourced health systems. For now, the problem is being addressed on a case-by-case basis, as decisions are made about whether or not particular medicines are affordable — though there is a danger that this will provoke public dissatisfaction with systematic efforts to deliver affordable healthcare.

Meanwhile, research which might instead help to reduce healthcare spend — for instance by stratifying patient populations to ensure that medicines are only delivered to those who are likely to benefit, or by identifying new uses for older and no-longer-profitable forms of treatment — goes relatively neglected. There is thus a need to rebalance research priorities so as to place greater emphasis on the kinds of innovation that would favour public health concerns over commercial interests. This will not be easy. For one thing, commercial actors in the personalised medicine arena are unlikely to support such a move. But more generally, as we have seen, the promissory economy of personalised medicine has evolved in such a way that public benefit is overwhelmingly identified with commercial success. Consequently, any attempt to reorient personalised medicine research in a way that better serves the public interest will require careful unpicking of that promissory economy, in order to distinguish those areas of work that can be effectively pursued through commercial partnerships, from those that require a conscious uncoupling of publicly funded medical innovation from the pervasive expectation of commercial profit. This will involve more than just demarcating hype from legitimate promise. It will require sustained reflection, self-criticism and often self-denial on the part of public research funders and the scientists they support.

## Related video

A video linked to this paper can be found at: http://www.healthcarevalues.ox.ac.uk/steve-sturdy-promise-and-hype-personalised-medicine
